# Hydroxyurea Use After Transitions of Care Among Young Adults With Sickle Cell Disease and Tennessee Medicaid Insurance

**DOI:** 10.1001/jamanetworkopen.2021.28971

**Published:** 2021-10-13

**Authors:** Joacy G. Mathias, Vikki G. Nolan, Lisa M. Klesges, Sherif M. Badawy, William O. Cooper, Jane S. Hankins, Matthew P. Smeltzer

**Affiliations:** 1 Department of Epidemiology, Gillings School of Global Public Health, University of North Carolina at Chapel Hill, Chapel Hill; 2 Division of Epidemiology, Biostatistics, and Environmental Health, School of Public Health, The University of Memphis, Memphis, Tennessee; 3 Department of Surgery, Washington University School of Medicine, St Louis, Missouri; 4 Division of Hematology, Oncology, Neuro-Oncology, and Stem Cell Transplantation, Ann & Robert H. Lurie Children’s Hospital of Chicago, Chicago, Illinois; 5 Department of Pediatrics, Vanderbilt University, Nashville, Tennessee; 6 Department of Health Policy, Vanderbilt University, Nashville, Tennessee; 7 Department of Hematology, St Jude Children’s Research Hospital, Memphis, Tennessee

## Abstract

This cohort study examines whether hydroxyurea use changes in young adults with sickle cell disease who are transitioning from pediatric to adult health care.

## Introduction

During transition from pediatrics to adult health care, young adults with sickle cell disease (SCD) face increased risk of severe morbidities and premature mortality.^1^ Sickle cell disease complications can be mitigated with hydroxyurea use.^2^ However, successful alleviation and prevention of SCD morbidities is contingent on initiation of and continued adherence to hydroxyurea therapy.^3,4^ With higher frequency of SCD-related complications among young adults, we examined the prevalence of hydroxyurea prescription among patients aged 18 to 25 years using statewide claims data. Understanding the patterns of hydroxyurea use in this patient population is key to potentially preventing the high burden of the disease accumulating among young adults.

## Methods

This cohort study was conducted with Tennessee Department of Health Institutional Review Board approval with a waiver of informed consent because only deidentified data were used for analysis. The study followed the Strengthening the Reporting of Observational Studies in Epidemiology (STROBE) reporting guideline. We obtained individual-level protected health information for all Medicaid claims of young adults with SCD during the study period (January 1, 2010, to September 30, 2015; analyzed from January 1, 2020, to December 31, 2020) and included those eligible for hydroxyurea prescription (eMethods in the Supplement). Young adults were classified into groups according to hydroxyurea exposure using medication possession ratio (MPR), which was defined as the total amount dispensed divided by the total number of days of study inclusion multiplied by 100, including not prescribed or never used (0%); low exposure (> 0 to ≤33.3%); medium exposure (>33.3% to ≤66.7%); and the reference group, high exposure (> 66.7%).^5^ Demographic covariates included age, region of the state, and sex. Clinical covariates included a visit to a hematologist in the year before the start of the study and genotype, grouped as hemoglobin (Hb) SS and HbSβ^0^ thalassemia, HbSC and HbSβ^+^ thalassemia, and other genotypes.

Odds ratios (ORs) with 95% CIs and Kruskal-Wallis tests were used to assess for an association between MPR and demographic characteristics and whether the ordered categories of MPR varied among patients aged 18 to 25 years. The level of statistical significance was set at a 2-sided *P* < .05. Statistical analyses were performed using the SAS software package, version 9.4 (SAS Institute Inc).

## Results

Of the 573 individuals aged 18 to 25 years (342 women [59.7%] and 231 men [40.3]; mean [SD] age at study start, 18.2 [3.8] years), 216 (37.7%) had at least 1 day of a filled hydroxyurea prescription. The prevalence of filled prescriptions were higher among men than women (OR, 2.0; 95% CI, 1.4-2.9), among those with a genotype of HbSS or HbSβ^0^ thalassemia than among those with HbSC or HbSβ^+^ thalassemia and other genotypes (OR, 36.9; 95% CI, 11.6-117.6), and among those who visited a hematologist in the year before the start of the study compared with those who did not (OR, 3.3; 95% CI, 2.2-5.1) (Table). The prevalence of filled prescriptions was somewhat higher among individuals aged 18 to 21 years than among those aged 22 to 25 years (OR, 1.3; 95% CI, 0.9-2.0) (Table). Hydroxyurea prescription fill uptake varied by region; individuals living in the east region were less likely to fill hydroxyurea prescriptions compared with the west (OR, 0.5; 95% CI, 0.3-0.8) (Table). Among 36 individuals aged 19 years, 38.9% had no MPR compared with 70.9% of the 99 individuals aged 23 to 25 years (Figure). MPR had a substantial downward pattern with increasing age, with a high MPR of 20% in those aged 18 years, 31% in those aged 19 years, 11% in those aged 20 and 21 years, and 7% in those aged 23 to 25 years (Kruskal-Wallis test, *P* = .02) (Figure).

**Table.** Prevalence of Hydroxyurea Use Among Young Adults Eligible for Hydroxyurea Prescription

Variable	At least 1 day hydroxyurea fill uptake, No. (%)		OR (95% CI)
	Yes (n = 216)	No (n = 357)	
Age at study start, y			
18-21	171 (79.2)	266 (74.5)	1.3 (0.9-2.0)
22-25	45 (20.8)	91 (25.5)	[Reference]
Sex			
Male	110 (50.9)	121 (33.9)	2.0 (1.4-2.9)^a^
Female	106 (49.1)	236 (66.1)	[Reference]
Region			
East	21 (9.7)	71 (19.9)	0.5 (0.3-0.8)^a^
Middle	53 (24.5)	71 (19.9)	1.1 (0.8-1.7)
West	142 (65.7)	215 (60.2)	[Reference]
Genotype			
HbSS or HbSβ^0^ thalassemia	213 (98.6)	235 (65.8)	36.9 (11.6-117.6)^a^
HbSC or HbSβ^+^ thalassemia and other genotypes	3 (1.4)	122 (34.2)	[Reference]
Visit to hematologist in year before study start			
Yes	70 (32.4)	45 (12.6)	3.3 (2.2-5.1)^a^
No	146 (67.6)	312 (87.4)	[Reference]

Abbreviation: OR, odds ratio.

^a^ Significant at α = .05.

**Figure.**
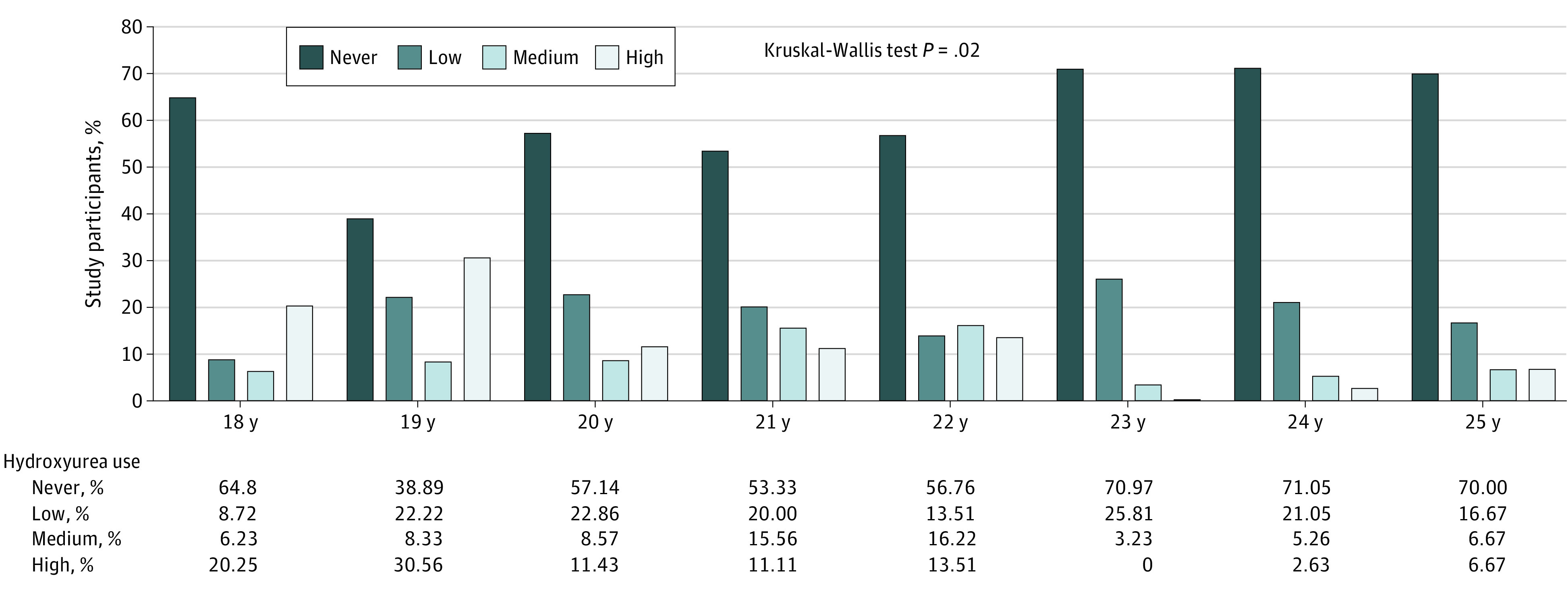
Categories of Hydroxyurea Fill Uptake Stratified by Age of Study Participants Never indicates that there was no prescription filled for hydroxyurea during the study period; low, more than 0 to 33.3% of time with a prescription filled for hydroxyurea; medium, more than 33.3% to 66.7% of time with a prescription filled for hydroxyurea; and high, more than 66.7% of time with a prescription filled for hydroxyurea (reference group).

## Discussion

With increasing age, hydroxyurea prescription fills decreased considerably among young adults with SCD and Medicaid insurance. Changing public insurance eligibility requirements may affect the ability of young adults with SCD to obtain a prescription for hydroxyurea. Women had a lower prescription uptake than men, possibly because of reproductive concerns.^6^ Visiting a hematologist was associated with substantially higher hydroxyurea prescription uptake. Implementing systematic transitioning programs for young adults with SCD, prescribing support for physicians, and providing access to specialized care centers may improve hydroxyurea use. Differences in hydroxyurea use by age, treatment by hematologist (pediatric and adult hematologists), and sex as well as the implications for health outcomes warrant further investigation. A limitation of this study was that using prescription refills as an indirect measure of hydroxyurea uptake can lead to possible misclassification.

## Notes

Supplement.
eMethods.
