# Supplementary material for: Hydroxyurea Use After Transitions of Care Among Young Adults With Sickle Cell Disease and Tennessee Medicaid Insurance
Source: JAMA Netw Open. 2021 Oct 13;4(10):e2128971. doi: 10.1001/jamanetworkopen.2021.28971 (PMC8515209; doi:10.1001/jamanetworkopen.2021.28971)
Supplement: Supplement. — eMethods. [file jamanetwopen-e2128971-s001.pdf]

Supplementary Online Content

Mathias JG, Nolan VG, Klesges LM, et al. Hydroxyurea use after transitions of care among young adults with sickle cell disease and Tennessee Medicaid insurance. *JAMA Netw Open.* 2021;4(10):e2128971. doi:10.1001/ jamanetworkopen.2021.28971

eMethods.

This supplementary material has been provided by the authors to give readers additional information about their work.

© 2021 Mathias JG et al. *JAMA Network Open*.

eMethods.

Study Population

Hydroxyurea eligibility was defined as HbSS and HbSβ^0 thalassemia genotypes with ≥3 episodes of severe vaso-occlusive event in the preceding 12-months from the start of cohort study period (for each individual). We also evaluated non-HbSS and HbSβ^0thalassemia genotypes with ≥3 episodes of severe pain in the preceding 12-months. Cohort members who were on hydroxyurea within the first 30 days of follow-up (January 1, 2010 and September 30, 2015) were considered as eligible. Furthermore, those younger than 18 years who transitioned to adulthood during the cohort study period and on hydroxyurea on their 18th birthday were considered to be eligible. For those younger than 18 years who transitioned to adulthood during the study period, and not on hydroxyurea on their 18th birthday, had to fulfill the hydroxyurea eligibility as defined for other adults. Once adults met guidelines for hydroxyurea, all subsequent person time were considered as meeting the guidelines. Upon fulfillment of hydroxyurea eligibility criteria, we identified the analytic population of YA between 18 to 25 years of age.

Study follow-up time for the analytic population began on the date of hydroxyurea eligibility fulfillment or the date they turned 18 years if already eligible. Study participants follow-up period ended at death, loss of enrollment in the health plan of >30 days (to account for brief administrative gaps), or the end of the study period (Sep 30, 2015), whichever occurred first. Participants were allowed to re-enter the cohort when they met study eligibility criteria again.

© 2021 Mathias JG et al. *JAMA Network Open*.

Exposure

Participants were classified according to their amount of exposure to hydroxyurea. Hydroxyurea was identified using NDC codes and depending on the availability of the information, patient medical records was used as a supplement to capture hydroxyurea dispensation. Medication use during hospitalization was not recorded in our data sources and hospitalization time was excluded from follow-up.

MPR was categorized into four groups: not prescribed/never user (0% MPR), low (>0 to <=33.3%), medium (>33.3% to <=66.7%), and the reference group, high user (>66.7%). This grouping allowed for a balanced distribution of MPR values among those with any hydroxyurea use in the dataset and to allow comparisons followed a convention from previous studies using a cutoff of 67%. [34,35] A study participant who had not filled a prescription for hydroxyurea in the preceding year by study start date was defined as a “never” hydroxyurea user.

© 2021 Mathias JG et al. *JAMA Network Open*.
